# Adam Opalski (1897–1963)

**DOI:** 10.1007/s00415-017-8704-8

**Published:** 2017-12-16

**Authors:** Liliana Rytel, Piotr Lech, Kamila Szymanska, Slawomir Gonkowski

**Affiliations:** 1 Department of Internal Diseases with Clinic, Faculty of Veterinary Medicine, University of Warmia and Mazury, Oczapowskiego Str. 14, 10-718 Olsztyn, Poland; 2 Agri Plus sp. Z o.o, Marcelinska Str. 92, 60-324 Poznan, Poland; 3 Department of Clinical Physiology, Faculty of Veterinary Medicine, University of Warmia and Mazury, Oczapowskiego Str. 13, 10-718 Olsztyn, Poland

Adam Opalski was born on November 26 (some sources say 28), 1897 in Olkusz, a town located about 40 km from Cracow in Poland. He attended elementary school in Kielce and middle school in Warsaw [1]. In 1917, Opalski started to study at the Medical Faculty of Warsaw University. He got a medical degree in 1924 and took a job in the internal diseases department at Baby Jesus Clinical Hospital, and a year later at the Warsaw Neurological Clinic [2].

Initially, Opalski worked as a volunteer and assistant. In the years 1928/29 and 1932/33, he went to Munich for an internship founded by the National Fund of Science and the Rockefeller Foundation [1]. In Germany, he worked at the laboratory of Professor Walther Spielmayer. During the internship Opalski studied the anatomy of the trigeminal ganglion under physiological conditions and during pathological states. However, his most important observations concerned altered glial cells occurring in the brain in Wilson’s disease. Opalski called these cells “outgrown giant glial cells” [3]. Interestingly, Opalski found these cells in tissues which were previously studied by Professors Alzheimer and Spielmayer, who did not see them [4].

Many years later, in 1965, professor Mossakowski, a student and co-worker of Opalski, called these “Opalski cells” in honour of their discoverer [5]. At present, this term is found in many textbooks of neurology and neuropathology.

During his stay in Munich, Opalski also wrote an assistant professorial dissertation entitled “Morphology and pathogenesis of inflammations within ependyma and subependymal glial cells” [4]. The defence of this dissertation took place in Warsaw in 1935. Opalski became the head of the Laboratory of Brain Histopathology at the Neurological Clinic in Warsaw, where he introduced new methods of brain labelling imported from Germany [4]. He was also a docent at the Department of Neurology of Warsaw University.

In 1939, when the Second World War broke out, Adam Opalski served in the Polish army as a medical officer, was taken prisoner and interned in Radom [1]. When Opalski was released from the prison camp in 1941, he worked in various Warsaw hospitals. In 1942, he became the head of Neurological Clinic and started the underground teaching of Polish medical students. During the Warsaw Uprising in 1944 the city was completely destroyed. Opalski saved the equipment of the Clinic, driving it away from Warsaw at risk to his life [4].

After the liberation of Warsaw, in February 1945 Opalski became head of Department of Neurology with Clinic at Warsaw University. Despite difficult post-war conditions, he organized student education and developed the activities of his department. He participated actively in the Polish Neurological Society and edited the journal “Polish Neurology and Psychiatry” [4].

At this time Opalski also worked as a scientist. In 1946, based on two clinical cases, he described the submedullary syndrome, which is now also known as Opalski’s sub-bulbar syndrome [6]. This disease is a rare neurological syndrome considered to be a variation of lateral medullary syndrome (Wallenberg’s syndrome) [7]. Since 1946 only a few cases of Opalski’s sub-bulbar syndrome have been described in the scientific literature [8]. This syndrome is caused by post-stroke ischemia in the area of branches of the posterior medullary artery and may be a result of various reasons including differences in the diameter of the vertebral arteries, dissection of the vertebral artery, or hypotonia secondary to spinocerebellar tract injury [7, 8]. Nevertheless, the most common cause of the syndrome is atherosclerosis [9]. The principal clinical features of Opalski’s sub-bulbar syndrome are as follows [7, 9]: ipsilateral hemiparesis and ataxia, Horner’s syndrome, hypoesthesia within the face, as well as alternate hypoesthesia for pain and temperature within trunk and contralateral limbs. In contrast to Wallenberg’s syndrome, during which no limb weakness is observed, a characteristic symptom of Opalski’s syndrome is hemiplegia [8]. It is ipsilateral to the changes in posterior medullary arterial territory because these changes are located caudal to the pyramidal decussation. This fact distinguishes Opalski’s syndrome from Babinski–Nageotte syndrome, where lesions are positioned before decussation and cause contralateral hemiparesis [7, 9].

Also, in the 1940s, Opalski went to the USA at the invitation of the Rockefeller Foundation, where he worked on electroencephalography (at that time it was relatively new) [4]. It should be pointed out that the first electroencephalograph imported to Poland was the gift for Opalski from the Rockefeller Foundation. After his return to Poland, Opalski continued his academic and scientific career. At that time he published numerous scientific papers and some academic manuals within the scope of neurology and neuropathology. The most important was “Histopathology of the nervous system” published in 1949, the first Polish book in this field, and “Vascular origin of diseases of the central nervous system and old age diseases” published in 1951 [4]. Moreover, Opalski worked on neurological symptoms during hypoglycemia, various aspects of multiple sclerosis, and neurological changes during syphilis [1, 2]. He also spent a lot of time teaching young clinicians.

He became a member of numerous scientific associations, including the Polish Academy of Learning in 1948, the Polish Academy of Sciences in 1948, the American Academy of Neurology in 1951 and the French Neurological Association in 1956 [4]. In 1954, Opalski received the title of full professor, and in 1955, he was awarded the Order of Polonia Restituta.

Unfortunately, at the most active period in his working life, Opalski had a stroke. After the disease he came back to work, but due to his deteriorating health he retired in 1958. The last years of his life Opalski spent in isolation and loneliness as a consequence of disease. Professor Adam Opalski died in Warsaw on November 3, 1963 at the age of 66 and was buried at Powązki Cemetery [4].

Adam Opalski was not only a talented and inquisitive scientist, but also a man of extraordinary personality. According to his students and co-workers, Opalski was a true “Renaissance man”, who was interested in various areas of life including classical music, art, nature and choreography [10]. These character traits and great commitment to scientific research caused Professor Adam Opalski to be called “the Chosen One of Science” [4].
